# Immune Response to Human Metapneumovirus Infection: What We Have Learned from the Mouse Model

**DOI:** 10.3390/pathogens4030682

**Published:** 2015-09-18

**Authors:** Nagarjuna R. Cheemarla, Antonieta Guerrero-Plata

**Affiliations:** 1 Department of Pathobiological Sciences, Louisiana State University, Baton Rouge, LA 70803, USA; E-Mail: ncheem1@tigers.lsu.edu; 2 Center for Experimental Infectious Diseases Research, Louisiana State University, Baton Rouge, LA 70803, USA

**Keywords:** human metapneumovirus, paramyxovirus, mouse model, immune responses

## Abstract

Human Metapneumovirus (hMPV) is a leading respiratory viral pathogen associated with bronchiolitis, pneumonia, and asthma exacerbation in young children, the elderly and immunocompromised individuals. The development of a potential vaccine against hMPV requires detailed understanding of the host immune system, which plays a significant role in hMPV pathogenesis, susceptibility and vaccine efficacy. As a result, animal models have been developed to better understand the mechanisms by which hMPV causes disease. Several animal models have been evaluated and established so far to study the host immune responses and pathophysiology of hMPV infection. However, inbred laboratory mouse strains have been one of the most used animal species for experimental modeling and therefore used for the studies of immunity and immunopathogenesis to hMPV. This review summarizes the contributions of the mouse model to our understanding of the immune response against hMPV infection.

## 1. Introduction

Human metapneumovirus (hMPV), belongs to the *Paramyxoviridae* family and represents the first human member of the genus *Metapneumovirus*. hMPV is a leading respiratory viral pathogen causing acute respiratory tract infection (ARTI) in young children, the elderly and immunocompromised individuals [1]. hMPV was first isolated in the Netherlands in 2001 from respiratory specimens of young children suffering with acute respiratory tract illness [2] and represents a major respiratory pathogen worldwide. Epidemiological studies show that hMPV is responsible for 5%–15% of pediatric hospitalizations for respiratory tract infections [3,4,5,6,7]. It induces clinical syndromes ranging from mild disease to more severe disease, with high fever, wheezing, severe cough, difficulty in breathing, tachypnea, bronchiolitis and pneumonia [8,9,10].

**Figure 1:**
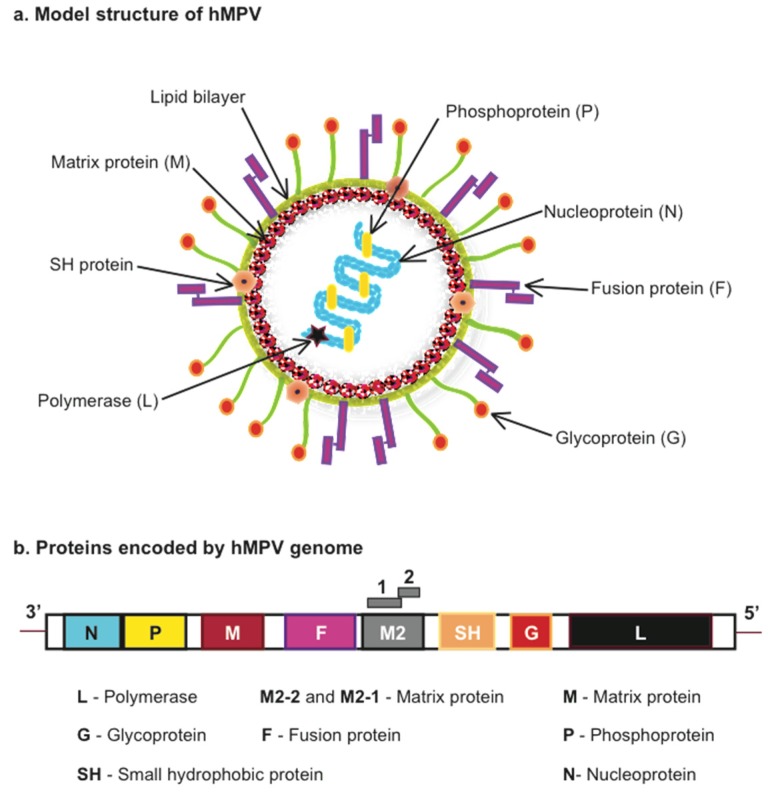
Model structure and proteins encoded by Human Metapneumovirus (hMPV). (**a**) hMPV model structure indicating viral proteins encoded by (**b**) the viral genome.

hHMPV is an enveloped, negative sense single-stranded RNA virus (Figure 1). Based on phylogenetic analysis, hMPV is classified into four genetic lineages, named A1, A2, B1 and B2 that divide into the A and B antigenic subgroups that belong to one serotype [11,12]. hMPV genome size is approximately 13,000 nt as it varies depending on the strain. Examples of the subgroup A indicate that the strain CAN97-83 is 13,335 nt and NL/00/1 is 13,350 nt, and for the subgroup B: CAN98-75 is 13,280 nt and NL/1/99 is 13,293 nt [11,13]. The hMPV sequence includes eight genes encoding nine proteins: nucleocapsid (N), phosphoprotein (P), matrix (M), second matrix (M2-1, M2-2), fusion (F), small hydrophobic (SH), attachment (G) and RNA-dependent RNA polymerase (L). The gene order in hMPV is represented as 3′-N-P-M-F-M2-SH-G-L-5′ (Figure 1). The attachment (G) and small hydrophobic (SH) genes are found to be highly variable while a high level of sequence conservation has been observed for the fusion (F) gene [13]. The G protein is a transmembrane surface glycoprotein, which initiates the virus-host cell membrane attachment and so considered as a key player in viral replication. The fusion (F) protein is required for the fusion of virus with host cell membrane and is capable of being accessed by neutralizing antibodies. The nucleocapsid (N), phosphoprotein (P) and RNA-dependent RNA polymerase (L) proteins along with M2 protein are involved in RNA synthesis [11,14,15].

Several animal models including mouse (see Table 1), cotton rat [16,17,18,19], hamster [20,21,22], ferret [20] and nonhuman primate models [20,23,24] have been established to date to study the immunopathology occurring after hMPV infection. Among them, the mouse model has provided considerable knowledge towards our understanding of the hMPV-host interaction. Thus this review focuses on the current knowledge of the immunity and immunopathology induced by hMPV in the experimental mouse model of infection.

**Table 1:** Different conditions for mouse infection with hMPV.

Mice Strain	Mice Age	(Group) Strain	Virus Dose	Refs.
BALB/c	F 6–8 week-old	(A) NL 00-01	3.3 × 10^5^ PFU	[25]
BALB/c	F 4–6-week-old	(A) C-85473	1.5 × 10^5^–10^8^ TCID_50_	[16,26,27,28,29,30,31,32]
BALB/c	F 6–8-week-old	(A) C4-CJP05	10^6^ PFU	[33]
BALB/c	F 4–6-week-old	(B) CAN98-75	0.8–1 × 10^6^ PFU	[29,34,35]
BALB/c	F 5–7 week-old	(A) NL/1/00	10^6^–10^7^ PFU	[20,36]
BALB/c	F 6–7 week-old	(B) NL/1/99	10^7^ PFU	[36]
BALB/c	F 6–10 week-old	(A) CAN97-83	10^6^–10^7^ PFU/TCID_50_	[30,37,38,39,40,41]
BALB/c	F 5–6 week-old	(A) CZ0107	10^6^ PFU	[42]
BALB/c	M 19 month-old	(A) CAN97-83	2 × 10^7^ geq	[43]
BALB/c	F 8–10 week-old	(A) D03-574	2 × 10^5^ PFU	[44]
C57BL/6	6–10 week-old	(A) CAN97-83	10^6^–10^7^ PFU	[38,45,46,47,48,49]
C57BL/6	F 6–12 week-old	(A) TN/94-49	0.6–1.5 × 10^6^ PFU	[50,51,52,53]
DBA/2	5–6 week-old	(A) TN/94-49	10^5.9^ PFU	[17]
SCID	F 6–8 week-old	(A) NL/1/00	6.5 × 10^6^ PFU	[54]

PFU = Plaque Forming Units; geq = genome equivalents; TCID50 = 50% tissue culture infective dose.

## 2. hMPV Infection in Mice

The experimental mouse model of hMPV infection has been established in several mouse backgrounds using different hMPV strains at diverse inoculum concentrations, as shown in Table 1.

Intranasal inoculation of mice with hMPV induces pulmonary inflammation characterized by interstitial inflammation and/or peribronchiolar and perivascular cellular infiltration [30,35,39,49], body weight loss with a peak of 15%–25% [16,25,32,34,41], altered respiratory function characterized by a significant increase in airway obstruction on day 5 after hMPV infection that could persist until day 21 [30], and lung viral titers that peak between day 3 to day 14 after hMPV infection [16,25,30,41].

However, some variations can be observed depending on the different experimental conditions. For instance, intranasal inoculation of BALB/c mice with hMPV CAN98-75 resulted in a biphasic lung viral replication with peaks at day 7 and day 14 [34,35] while infection of BALB/c mice with any other hMPV strain led to a one-peak only of viral titer on or before day 5 after infection (Table 2). Based on the data from the reports included in Table 2, BALB/c mice appear to be more permissive than C57BL/6 mice. Although, shedding of infectious virus beyond the recovery phase has been rarely reported [34], detection of hMPV transcripts have been found at day 154 [30] and 180 [34] after infection, suggesting that hMPV could persist in the lung of infected animals since hMPV infection has been characterized as a localized infection affecting just the airways but no other organs [35].

**Table 2:** Mouse susceptibility and permissibility to hMPV.

Mice Strain	Virus Strain	Virus Inoculum	Peak Viral Titer	Ref.
BALB/c	NL/1/00	3.3 × 10^5^ PFU	Day 4 (Log_10_ 2.37 PFU/g)	[25]
BALB/c	CAN97-83	10^7^ TCID50	Day 4 (10^5^ TCID_50_/g)	[41]
BALB/c	C85473	1.5 × 10^5^ TCID_50_	Day 6 (~10^4^ TCID_50_/lung)	[26]
BALB/c	C85473	1 × 10^8^ TCID_50_	Day 5 (7 × 10^6^ TCID_50_/lung)	[30]
BALB/c	C85473	1 × 10^8^ TCID_50_	Day 5 (1.92 × 10^7^ TCID_50_/g)	[16]
BALB/c	C85473	5.8 × 10^5^ TCID_50_	Day 5 (~10^5^ TCID_50_/g)	[32]
BALB/c	NL/1/00	1.5 × 10^5^ PFU	Day 5 (5.1 × 10^5^ PFU/g)	[55]
BALB/c	D03-574	2 × 10^5^ PFU	Day 4 (~10^3.6^ PFU/lung)	[44]
C57BL/6	CAN97-83	5 × 10^6^ PFU	Day 5 (10^4.9^ PFU/g)	[46]
C57BL/6	TN/94-49	1 × 10^6^ PFU	Day 5 (~4.7 Log_10_ PFU/g)	[53]
C57BL/6	CAN97-83	1 × 10^7^ PFU	Day 5 (~4.1 Log_10_ PFU/g)	[47]
C57BL/6	TN/94-49	6 × 10^5^ PFU	Day 5 (~4.2 Log_10_ PFU/g)	[51]

## 3. Lung Antiviral and Inflammatory Responses

### 3.1. Innate Immunity

Innate immune responses to viral infections in the lung serve as the first line of defense and it is activated upon recognition of the pathogen by immune cells in the respiratory tract. The cellular barrier constituting neutrophils, macrophages, natural killer (NK) cells and dendritic cells (DC) play a key role in the innate immune responses, which is triggered by the recognition of pathogen associated molecular pattern (PAMP) by cell receptors called pattern recognition receptors (PRRs) expressed in most cells of the respiratory tract. These pattern recognition receptors are broadly classified into membrane bound Toll-like receptors (TLRs), C-type lectin receptors (CLR), cytoplasmic RIG-I-like receptors (RLRs) and nucleotide-binding oligomerization domain (NOD)-like receptors (NLRs) [56]. The recognition of viral PAMPs by the cellular PRRs initiate the activation of signaling pathways leading to the production of cytokines and chemokines by the cells in the respiratory tract, that in turn regulate the inflammatory and immune responses in the infected host.

#### 3.1.1. Pattern Recognition Receptors and Signaling Pathways

We have recently demonstrated the importance of the RLR helicase melanoma differentiation-associated gene 5 (MDA5) in the type I (α/β) and type III (λ) interferon (IFN) production by hMPV infection [45]. In a model of MDA5-deficient mice (C57BL/6J background) infected with hMPV CAN97-83, the lack of MDA5 resulted in a decreased viral clearance, enhanced disease severity and pulmonary inflammation, and was necessary for the production of IFN-α/β and IFN-λ2/3. Moreover, MDA5 regulated the production of cytokines and chemokines in response to hMPV, demonstrating the critical role MDA5 plays in the control of hMPV-induced disease [45]. Downstream of the MDA5 signaling pathway, this helicase interacts with the adaptor molecule IFN-promoter stimulator 1 (IPS-1) at the mitochondrial membrane in order to induce the expression of cytokines [57]. In that regard, studies in neonatal IPS-1 deficient mice (C57BL/6 background) have shown that the absence of IPS-1 led to an increased viral load and decreased production of IFN-β and IFN-λ2/3 at day 1 after hMPV infection [58], indicating that IPS-1 contributes to the antiviral response and hMPV clearance. Moreover, similar IFN response to hMPV infection in the absence of IPS-1 has been reported in adult mice [59]. Thus, these findings confirm the key role for the MDA5 and IPS-1 signaling pathway in the antiviral response against hMPV infection.

On the other hand, data of hMPV infection in C57BL/10ScSnJ Toll-like receptor 4 (TLR4) deficient mice have shown that absence of TLR4 resulted in a decreased inflammatory response, disease severity, as well as IFN-α/β and cytokine production [48]. In line with those data, the lack of myeloid differentiation protein response 88 (MyD88), an essential adaptor molecule for TLR’s (except TLR3), led to a reduced lung inflammation and disease severity compared to wild type mice. The absence of MyD88 also impaired the production of cytokines and chemokines and the recruitment of DC, CD4 and CD8 T cells into the lungs of infected mice [47]. Collectively, these studies indicate that TLR4 and MyD88 are key molecules that regulate the hMPV-induced pulmonary inflammation and disease pathology in mice.

Signaling via PRRs ultimately leads to the activation of the transcription factors interferon (IFN) regulatory factors (IRFs), which induce the expression of the interferons and cytokine responses. Data in C57BL/6 mice have demonstrated that the expression of both IRF3 and IRF7 were necessary for the production of IFN-α/β [45]. In agreement with these results, in hMPV-infected C57BL/6 neonatal mice, both IRF3 and IRF7 were necessary for the expression of IFN-α4 and IFN-β. Moreover, the absence of both IRF3 and IRF7 exacerbated the Th1, Th2, and Th17 lymphocyte responses as well as the recruitment of neutrophils, eosinophils, NK and NK T cells in response to hMPV infection [58]. Similarly, the production of IFN-λ2/3 after hMPV infection was regulated by the expression of IRF-7 in adult [49] and neonatal [58] mice. However, the expression of IRF-3 was necessary for the production of IFN-λ2/3 in neonatal mice [58] but it was dispensable when the IFN-λ2/3 was induced by hMPV in adult mice [49], suggesting that the activation of the IFN-λ response by hMPV in adult and young mice is differentially regulated by IRF-3 and IRF-7 expression. Interestingly, hMPV has also been reported to inhibit the IFN responses [39,49]. Studies in BALB/c mice have demonstrated that hMPV infection inhibits the poly-ICLC- (synthetic dsRNA, TLR3/RIG-I/MDA5 agonist) and CpG-ODN- (TLR9 agonist) induced IFN-α production [39], suggesting that hMPV infection is able to inhibit the activation of RLRs and TLRs *in vivo*. In addition, recent data have shown that hMPV G protein inhibits the production of IFN-λ2/3 in BALB/c mice after hMPV infection, at least through the interference with the RIG-I/MDA5 pathway [49].

Based on the reported observations described above, hMPV-induced immune response is regulated by the activation of selected PRRs. It appears that hMPV infection activates TLRs to induce an inflammatory response while it subverts RLRs to alter the antiviral responses via the inhibition of interferons. This immune subversion is attributed to the expression of hMPV G protein. Taken together, experimental evidence demonstrates that hMPV is able to activate and subvert antiviral signaling pathways, likely through different mechanisms. However, unresolved pathways involved in activation or subversion of hMPV induced immune response, need further elucidation. A detailed understating of hMPV induced recognition and signaling cascades is crucial to developing effective therapeutics and vaccine strategies.

#### 3.1.2. Cytokine Production

hMPV is known to induce in humans a profile of cytokines distinct to other respiratory viruses such as respiratory syncytial virus (RSV), and influenza virus [60]. Although very scarce, studies comparing hMPV and RSV infection are clinically relevant as RSV is the closest related human paramyxovirus to hMPV [11]. In fact, symptoms between RSV and hMPV are indistinguishable, ranging from mild cold-like symptoms to more severe clinical manifestations like bronchiolitis or severe pneumonia that require hospitalizations [3,7,61]. However, some aspects of the immune response elicited by these two viral pathogens are distinct. This was demonstrated by the analysis of nasal washes from hospitalized infants showing that hMPV infection induced significantly lower amounts of proinflammatory cytokines including IL-12, IL-6, IL-8, TNF-α and IL-1β compared to RSV infection [60], suggesting that hMPV is a poor inducer of inflammatory cytokines compared to RSV in infected infants. In line with these data, research in the mouse model resembled the observation in human studies. Using BALB/c mice infected with hMPV (CAN97-83) and compared to RSV (A2) side-by-side, hMPV induced a weaker response of proinflammatory cytokines (IL-1α, IL-1β, IL-6, TNF-α, G-CSF) and regulatory cytokines (IL-10, IL-12p70, IL-17). However, hMPV induced a stronger response of IFN-α, GM-CSF, IL-18, CXCL1 (KC) and a sustained production of IL-12p40 [37]. In contrast to this work, a study conducted in BALB/c mice using a clinical hMPV isolate (D03-574) induced significantly higher levels of TNF-α, IL-6 and MCP-1 compared to RSV (A2) at day 4 and 7 post infection [44]. The discrepancies between these two studies in mice could be due to the use of different virus strains and virus stock preparations.

The effect of hMPV on the IFN response has been further confirmed since experimental observations indicated that hMPV induced a stronger response of IFN-β and IFN-λ2/3 when compared to RSV infection in BALB/c mice [49]. However, levels of IFN-γ were induced similarly by hMPV and RSV-infected BALB/c mice [44]. Additional data have also demonstrated the capacity of hMPV to induce several cytokines in the lung, where a significant induction of CCL2 (MCP-1) and CXCL1 (KC) on day 1 and IFN-γ, CCL5 (RANTES), CCL3 (MIP1α), and IL-4 on day 5 after hMPV infection has been observed [16,25]. Overall, these findings suggest that hMPV infection induces a unique profile of cytokines and chemokines in the lung of infected mice.

The regulatory effects that lung cytokines and chemokines exert in hMPV-induced disease are still largely unexplored. In that regard, IL-12p40, an induced cytokine during hMPV infection that remains sustained after the resolution of the disease [37] has been shown to be critical to control disease severity by regulating cytokine production, inflammatory response and mucin production in the lung. Using IL-12p40-/- mice infected with hMPV, showed an increased goblet cell formation, increased mucin gene expression in the airways and decreased lung function. IL-12p40 was found to specifically regulate the expression of IFN-γ, IL-6, CXCL10 (IP-10), CCL11 (eotaxin), CXCL1 (KC, IL-8 homolog) and CCL2 (MCP-1) in mice infected with hMPV [46]. Furthermore, the level of expression of inflammatory cytokines after hMPV infection appears to be altered in aged animals. For instance, TNF-α levels were decreased ~7-fold in 19 moth-old hMPV-infected mice when compared to 4–6 week-old animals [43] while IL-6 was increased in 18–19 month-old mice when compared to 6–8 week old mice [62]. Also, hMPV infection alters the cytokine response to opportunistic bacterial infection in the lung. Prior hMPV infection exacerbated the levels of TNF-α, IFN-γ, IL-1α, IL-1β, IL-6, IL-12p40, IL-12 p70, IL-9, IL-10, IL-13, KC, G-CSF, GM-CSF, MCP-1 and MIP-1α in *Streptococcus pneumonia*-infected mice and predisposed those animals to severe pneumococcal infection [26]. The described cytokine patterns induced by hMPV infection are crucial to understanding the underlying mechanisms in activation of the innate and adaptive immune responses as well as the initiation and resolution of the inflammatory response and lung viral clearance. However, the role of these cytokine pathways in promoting and modulating inflammation and host immune responses in hMPV infection are still largely unknown. The use of genetically modified mice will represent a critical tool to answer these relevant questions.

#### 3.1.3. Dendritic Cells

Dendritic cells (DC) are professional antigen-presenting cells within the immune system. Respiratory tract dendritic cells are present within airway epithelium, submucosa and associated lung parenchymal tissue under resting conditions [63]. Their strategic localization at the site of pathogen entry makes them particularly susceptible to initial viral invasion. After detection, uptake and degradation of viruses, DC initiate immune responses via the secretion of interferon (IFN), chemokines and proinflammatory cytokines, as well as the upregulation of a variety of costimulatory molecules and receptors, a process globally known as cell maturation. After maturation, DC efficiently present antigens and initiate adaptive immune response by migrating into lymph nodes (LN) to activate the virus-specific T cell response [32]. To date, there have been at least three major subsets of murine lung DC described. These include plasmacytoid DC (pDC), the myeloid DC (also known a conventional DC, cDC), and the interferon-producing killer dendritic cells (IKDC). DC have been reported to participate in the innate and adaptive immune response to hMPV infections, indicating their critical role in the antiviral immunity to this virus. Dendritic cells are susceptible to hMPV infection *in vitro* [64] and *in vivo* [40,65]. In fact, hMPV activates mouse lung DC, and induces the upregulation of costimulatory molecules and the secretion of several cytokines including IL-6, IFN-α, IFN-β and TNF-α [40]. hMPV infection also induced the recruitment of pDC and IKDC which peaked by day 8 after infection. The predominant subset recruited to the lung corresponded to cDC, and this remained the highest subset for at least 18 days, beyond the acute phase of infection. CD103+ cDC substantially decreased until three weeks after infection and returned to basal levels by week 8. Differential production of cytokines by murine lung pDC and cDC infected with hMPV was also observed. More interestingly, hMPV infection reduced the capacity of lung cDC to stimulate T cell responses [40], which is in line with some reports *in vitro* using human DC that indicate that hMPV alters their capacity to activate T cells [64,66].

#### 3.1.4. Alveolar Macrophages

Alveolar macrophages (AMs) are known to be the first line of defense against respiratory pathogens [67]. They reside in the pulmonary alveolus and survey the exposed airways to contribute to the innate host defense against inhaled insults [68]. They are essential source of immunomodulatory cytokines for host responses against lung infections and their depletion results in impaired host response [67,69,70]. In fact, recent work has demonstrated that AMs differentially control the antiviral response and airway inflammation in hMPV infection when compared with RSV [69]. Using a BALB/c mouse model, AMs were depleted using clodronate liposomes (L-CL_2_MBP) prior to hMPV infection. Depletion of AMs altered the hMPV-induced disease since there was a reduced body weight loss, lung viral titer, decreased lung inflammation and airway hyperresponsiveness (AHR). Moreover, the recruitment of CD4+ T lymphocytes was significantly decreased following AM depletion. AMs are sources of pro inflammatory cytokines and chemokines. In line with this, depletion of AMs resulted in significantly lower level of cytokines including IL-1α, IL-1β, TNF-α, IL-6, GM-CSF, G-CSF, CCL4, IFN-α and IFN-β. However, their depletion also induced an increased release of CCL3, CCL5, and IL-12p40 after hMPV infection [69]. Thus, the results of this study indicate that the presence of alveolar macrophages regulate and contribute to the hMPV-induced disease.

#### 3.1.5. Natural Killer Cells

Another component of the innate immune system are the natural killer (NK) cells, which are lymphocytes that respond to malignant tumors and intracellular pathogens including viruses. Studies conducted by Alvarez *et al.* demonstrated that NK cells have a leading role in controlling hMPV viral clearance [36]. Depletion of NK cells with anti-CD49b/Pan-NK cell monoclonal antibody in BALB/c mice resulted in increased lung viral titers on days 7, 28 and 60 after infection compared to NK cell competent mice. In contrast, work reported by Wen S. *et al.* in C57BL/6 mice have demonstrated that NK cells do not contribute to hMPV clearance [51]. Lung NK cell numbers in infected mice were, however, increased as early as day 1 after hMPV infection and peaked on day 3 compared to mock infected mice. Moreover, hMPV infection induced activation of lung NK cells, as indicated by the upregulation of CD69. However, depletion of NK cells using the anti-NK1.1 antibody did not result in changes in lung viral titers, lung histopathology, or the numbers of CD4+ and CD8+ T lymphocytes. Suggesting that, NK cells do not play a significant role in the host responses against hMPV, and that the clearance of the viral infection requires different set of immune components *in vivo*. The discrepancies between these two studies could be attributed to the use of different experimental conditions, as detailed above. Thus, further work to fully define the role of NK cells in hMPV infection is warranted.

### 3.2. Adaptive Immunity

Cell mediated immunity serves as an important barrier in the multi-step paradigm of immune responses to pathogenic mechanisms. These responses function mainly by activation of cytotoxic T-lymphocytes to induce apoptosis of virus-infected cells or by activating T helper cells to stimulate other immune cells such as macrophages, B cells and NK cells and aid in the production of distinct cytokine profiles to induce intercellular communication. Experimental evidence with clone-specific induction of cytotoxic T cells [38] and experimental models with T cell depletion studies [34,38,41] demonstrate the essential role of T lymphocytes in immune surveillance and protection in hMPV infection.

Characterization of the T cell response against this virus has indicated that hMPV results in an accumulation of virus-specific cytotoxic CD8+ T cells (CTL) in the lungs 7 days after infection but not in regional lymph nodes or spleen. However, a strong memory response can be recalled from the spleen at 21 days post infection [71]. Though both CD4+ and CD8+ T cells act synergistically and play an indispensable role in both inflammatory responses and anti-viral immunity, they have been found to induce different profile of cytokines after hMPV infection [35]. During primary infection, depletion of either of the two T cell subsets, or in fact both of them, caused reduced inflammation and body weight loss in hMPV-infected mice but were required for viral clearance. These data suggest that primary hMPV infection induces lung disease mediated, in large extent, by T cells while T cells are also necessary for the clearance of primary hMPV infection [41]. Regarding the regulation of the T cell response during hMPV infection, a recent study by Hastings *et al.*, showed that type I IFN signaling is essential for the development of functional hMPV specific CD8 T cells in the lungs using IFN-α receptor deficient C57BL/6 mice [53]. Moreover, in aged mice, CD4+T cells appear to play an important role in the exacerbated hMPV-induced disease. As demonstrated in 18-19 month-old BALB/c mice which showed a significant increased numbers of IL-4-producing CD4+ T cells but no change in the CD8+ T cell numbers when compared with younger mice [62], suggesting a Th2 skewing response in older mice after hMPV infection.

As for the role of T cells in hMPV reinfection, concurrent depletion of both CD4+ and CD8+ T cells led to a decreased airway hyperresponsiveness (AHR) [41]. However, depletion of CD4+ T cells alone during hMPV reinfection, unlike in CD8+ T cell-depleted mice, led to a defective antibody response. Nevertheless, CD4+ T cell-depleted mice had undetectable infectious virus after hMPV challenge and were protected from clinical disease, indicating that protection can be provided by an intact CD8+ T cell response [41]. Interestingly, recent observations indicate that the CD8+ T cell response is impaired during hMPV infection and reinfection and that phenomenon appears to be regulated by the expression of the inhibitor receptor programmed death-1 (PD-1) and programed death ligand-1 (PD-L1) [52,72,73]. These findings indicate that a defective CD8+T cell response contributes to hMPV reinfection. Whether this effect characterizes the commonly observed hMPV reinfection in humans warrants future research.

The understanding of the T cell response induced by hMPV vaccine candidates has found their initial steps using the mouse model. The induction of CD8+ cytotoxic T cells by peptide immunization in mice has proven to be protective against hMPV challenge in reducing viral load and lung histopathology [38]. Likewise, immunization with Bacillus Calmette-Guerin (BCG) strains expressing hMPV-phosphoprotein effectively induced a protective response which was mediated by a Th1 T cell response [42]. Immunization with hMPV F-bearing virus-like particles (VLP) was also able to stimulate an hMPV specific CD8+ T cell response and protected lungs from infection after hMPV challenge [50]. Further experimental studies in non-human primates and/or clinical trials are warranted in order to validate the immunological observations in the mouse model towards vaccine development.

## 4. Conclusions

The experimental mouse model represents a valuable tool for *in vivo* research on hMPV infection and has provided important information regarding the hMPV-induced disease and detailed aspects of the immune response induced by hMPV infection. Although, inherent limitations are observed in the mouse model when data are extrapolated to the natural human infection, due to the availability of several gene deficient mice strains and multiple murine specific antibodies, it provides a valued experimental small animal model that allows answering critical questions that are necessary to our better understanding of the immune response and disease pathogenesis of hMPV.
